# Novel Antiviral Agents: Synthesis, Molecular Modelling Studies and Biological Investigation, 2nd Edition

**DOI:** 10.3390/v17050601

**Published:** 2025-04-23

**Authors:** Simone Brogi

**Affiliations:** 1 Department of Pharmacy, University of Pisa, Via Bonanno 6, 56126 Pisa, Italy; simone.brogi@unipi.it; Tel.: +39-050-2219613; 2 Bioinformatics Research Center, School of Pharmacy and Pharmaceutical Sciences, Isfahan University of Medical Sciences, Isfahan 81746-73461, Iran

After the success of the Special Issue entitled “*Novel Antiviral Agents: Synthesis, Molecular Modelling Studies and Biological Investigation*” (https://www.mdpi.com/journal/viruses/special_issues/novelantiviral_agents, accessed on 8 April 2025) which closed in June 2023 [1], a second edition of the abovementioned Special Issue was launched in late 2023 as a continuation of the investigation of this relevant topic. In fact, because of the increased attention of viruses with pandemic potential (i.e., severe acute respiratory syndrome coronavirus 2 (SARS-CoV-2; COVID-19), Lassa and Crimean-Congo hemorrhagic fevers, Ebola and Marburg virus disorders, Middle East respiratory syndrome (MERS), severe acute respiratory syndrome (SARS), Zika virus, Rift Valley fever, henipavirus (Nipah and Hendra viruses), and Disease X), the World Health Organization (WHO) continues to recommend investing in antiviral research to develop effective broad-spectrum antiviral agents, diagnostic tests, and vaccines that could be useful for the next potential pandemic scenario [2,3]. In this context, each effort that allows us to gain knowledge of viruses’ behavior, improve the detection of viruses and their variants, and the development of therapeutic agents can be useful in preparing for potential outbreaks.

Accordingly, advancements in computer-based techniques, organic synthesis, and molecular biology can facilitate the identification of potential antiviral agents in a short period. The second edition of this Special Issue focused on this matter, publishing four research articles and one review article. This Editorial note summarizes papers published in this Special Issue.

The first paper was authored by Desmarets and collaborators. They identified four promising natural products with antiviral properties. In particular, they isolated 42 natural products from lichens and symbiotic organisms (fungi and bacteria) and characterized them as antiviral agents by screening against human coronavirus HCoV-229E using two different cellular systems (Huh-7 and Huh-7/TMPRSS2 cells). Interestingly, four natural products showed significant activity in the antiviral assays. In particular, vulpinic acid, perlatolic acid, emodin, and chloroatranol (Figure 1) inhibited HCoV-229E replication with IC_50_ values of 14.58, 16.42, 59.25, and 68.86 µM, respectively. Furthermore, the selected compounds did not exhibit toxicity at the effective concentration.

The authors performed kinetic analysis and determined that natural products exert antiviral potential by inhibiting virus replication. Considering these encouraging results, the authors tested the compounds against SARS-CoV-2 using a Vero-81-derived clone cell system with a GFP reporter probe. Among the four tested compounds, perlatolic acid exhibited the most significant dose-dependent anti-SARS-CoV-2 activity with no toxicity issues (IC_50_ = 31.9 μM). In summary, the authors confirmed that natural products represent a relevant source of bioactive compounds, including molecules with antiviral activity. Further studies are expected to properly characterize the mechanism of action and to investigate perlatolic acid as a potential broad-spectrum antiviral agent [4].

In the second paper, Yazdani and coworkers employed a series of computer-based techniques, mainly based on molecular docking and molecular dynamics (MD) simulations, to identify potential SARS-CoV-2 NSP3 Mac1 domain inhibitors. In particular, a high-throughput virtual screening campaign was conducted using Molegro Virtual Docker software, the three-dimensional structure of the macrodomain of SARS-CoV-2 NSP3 Mac1 (PDB ID: 6W02), and a chemical library available from the National Cancer Institute (NCI) that contains over 200,000 molecules that were experimentally proven by the Developmental Therapeutic Program (DTP) platform to possess some biological activity. Top-ranked compounds were selected based on MolDock and Re-Rank scores. In addition, the drug-likeness of the selected molecules was assessed using the SwissADME webserver. Finally, the binding stability and affinity of the top-ranked molecules within the selected binding site of the NSP3 Mac1 domain were evaluated by performing MD simulation experiments using Desmond software and applying the MM/GBSA method. These subsequent computational filters allowed for the selection of four compounds as potential NSP3 Mac1 domain ligands with relevant binding energies (ΔG_bind_) and favorable drug-like profiles. In particular, NSC-358078, NSC-287067, NSC-123472, and NSC-142843 (Figure 2) were selected for further characterization to develop innovative antivirals against SARS-CoV-2 [5].

In an interesting work, Wang and colleagues developed a novel cell-based model in which SARS-CoV-2 replication can be induced. In particular, SARS-CoV-2 directly damages cells, making it difficult to create cell culture models with low cell killing effects to study the replication process. For example, the use of systems involving a DNA vector-based replicon system that employed only the cytomegalovirus (CMV) promoter to produce a recombinant viral genome containing reporter genes led to the development of drug resistance and cytotoxicity, hindering the development of the model. In this study, the authors described a new cellular culture system in which SARS-CoV-2 replication is triggered by the *Cre*/*LoxP*-mediated DNA recombination process. A specially designed SARS-CoV-2 transcription unit was inserted into a bacterial artificial chromosome (BAC) plasmid. In addition, removal of the viral spike protein gene and substitution of the nucleocapsid gene with a reporter gene were implemented to boost biosafety. A non-native DNA sequence was introduced into NSP1 as a regulatory element and removed after a specific DNA recombination process involving *Cre*/*LoxP* sites, followed by RNA splicing. The transcription unit was integrated into the host cell’s chromatin using the PiggyBac transposon strategy, resulting in a stable cell line that stimulated the replication of recombinant SARS-CoV-2 RNA (Figure 3). The model was sensitive towards the potential antiviral agents forsythoside A and verteporfin. In summary, a novel, controllable SARS-CoV-2 replicon cell model with improved stability was developed to deepen our understanding of the virus’s replication and disease-causing mechanisms and to streamline the testing and evaluation of potential anti-SARS-CoV-2 antiviral agents [6].

The last published research article authored by Espíndola focused on dengue virus (DENV), which has shown an increased incidence in the interested population. DENV fever, also known as break-bone fever, is a viral disease transmitted to humans through mosquito bites (*Aedes aegypti* and *Aedes albopictus*) mainly in tropical and subtropical climates. Remarkably, in some cases, DENV causes severe symptoms that require hospitalization for proper care and can pose a threat to life. Currently, there is no established treatment for DENV infection, and thus patients are typically administered pain relief medication. In this context, the development of antivirals that can reduce virus transmission is urgently needed. In the work enclosed in this Special Issue, the author explored the possible application of flavones with anti-DENV activity in specific nanoparticles such as single-walled carbon nanotubes (SWCNTs). In particular, baicalein, tropoflavin, and luteolin (Figure 4) were evaluated for their potential interaction with DENV E-3 protein using computer-based techniques based on molecular docking and MD simulation. Computational studies were conducted considering the flavonoid binding sites located between domain I of chain B and domain II of chain A of DENV E-3 protein (PDB ID: 1UZG). The analysis revealed the considered natural products in complex with DENV E-3 interacted by H-bond, π-π stacking, and π-cation interactions with slight differences in binding energies, as calculated by AutoDock software. In addition, the stability of the binding of the selected compounds was assessed by MD simulation studies (Desmond software, version 2021.4) using the ligand/protein complexes obtained by the molecular docking studies. MD simulation studies conducted for 100 ns (replicated for 500 ns) indicated that the binding modes for these compounds were quite stable, with the capability of maintaining the main interactions for the duration of the simulation. Moreover, SWCNTs were generated using the Nanotube Modeler program to load natural products with potential anti-DENV activity. Natural products/SWCNTs were used for molecular docking studies to evaluate the possibility of binding to DENV E-3 protein. The results showed that natural products/SWCNTs could interact with domain II of the DENV E-3 protein with favorable binding energies. This approach is interesting considering the low bioavailability and difficulty in employing natural products in clinical practice. In fact, because of the significant technological advances in generating and utilizing nanoparticles, the use of SWCNTs in antiviral drug discovery can increase the success rate of discovering potential antiviral agents, thereby improving their delivery [7].

In this Special Issue, an interesting review article authored by Heffner and Rouault was published. It is undeniable that coronaviruses possess significant potential to inflict substantial harm and cause severe symptoms in humans. Unfortunately, due to the safety measures required to research highly virulent coronaviruses, Biosafety Level 3 (BSL-3) laboratories may be somewhat inadequate, with equipment that only meets the most fundamental necessities. It is worth mentioning that research on highly pathogenic coronaviruses is crucial for assessing the effectiveness of antiviral treatment before drug approval. In this context, in the last two years, along with the decrease in funding for coronavirus research and the difficulty of developing reliable cellular or animal model systems, research on SARS-CoV-2 has experienced a dramatic decrease. Accordingly, studying endemic coronaviruses offers a chance to test and evaluate antiviral therapies while minimizing biosafety risks, allowing for new discoveries to be made. In fact, endemic coronavirus research conducted in BSL-2 laboratories has now reached a new level of capability. Research into endemic human coronaviruses is expected to increase significantly and ultimately lead to a greater understanding of coronavirus biology and host cell biology, as well as improved treatments. In the presented review article, one alternative and popular strategy in antiviral research that involves the use of less virulent endemic coronaviruses as safer alternatives is described in depth to gain a deeper understanding of coronavirus biology. The authors compared human endemic coronaviruses to their more virulent counterparts and provided details on the latest developments in the cultivation of endemic coronaviruses (HCoV-229E, HCoV-OC43, HCoV-NL63, and HCoV-HKU1), which can serve as safer alternatives to the more pathogenic coronaviruses (SARS-CoV, MERS-CoV, SARS-CoV-2) used in research studies (Figure 5). This development is expected to result in significant advancements in antiviral research, a deeper understanding of host and viral biology, and enhanced insight into the circumstances under which viruses become endemic, a crucial consideration that will influence existing policies and treatment protocols for SARS-CoV-2 globally [8].

Finally, I extend my sincerest appreciation to the *Viruses* Editorial staff, all contributors, including authors and co-authors, for their substantial input into this Special Issue, as well as to the reviewers who meticulously assessed the submitted content. The success of this Special Issue can be attributed to the collective impact of these collaborative initiatives. It is hoped that this Special Issue will facilitate the development of effective antivirals and serve as a significant body of knowledge and a motivating force for researchers and students. This Special Issue is freely available and can be accessed through the following link: https://www.mdpi.com/journal/viruses/special_issues/0MGVEU0B48.

## Figures and Tables

**Figure 1:**
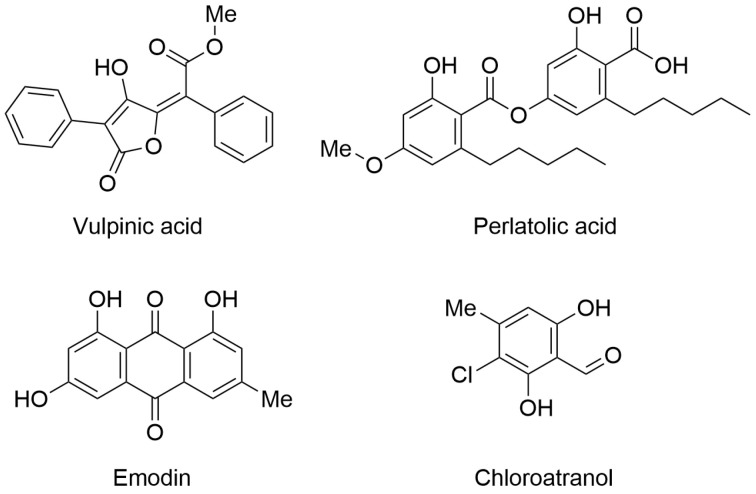
Chemical structures of lichen-derived compounds with antiviral profile. Vulpinic acid (PubChem ID 54690323), perlatolic acid (PubChem ID 174857), emodin (PubChem ID 3220), and chloroatranol (PubChem ID 6426722).

**Figure 2:**
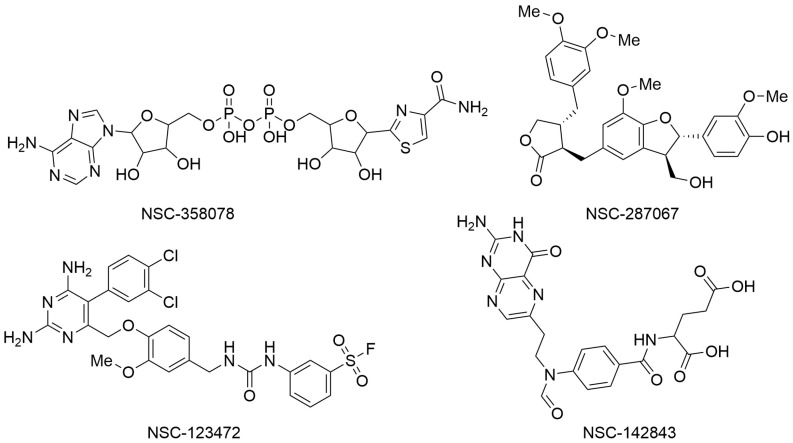
Chemical structures of the molecules identified as potential NSP3 Mac1 domain ligands. NSC-358078 (PubChem ID 13306556), NSC-287067 (PubChem ID 46173977), NSC-123472 (PubChem ID 420428), and NSC-142843 (PubChem ID 135500379).

**Figure 3:**
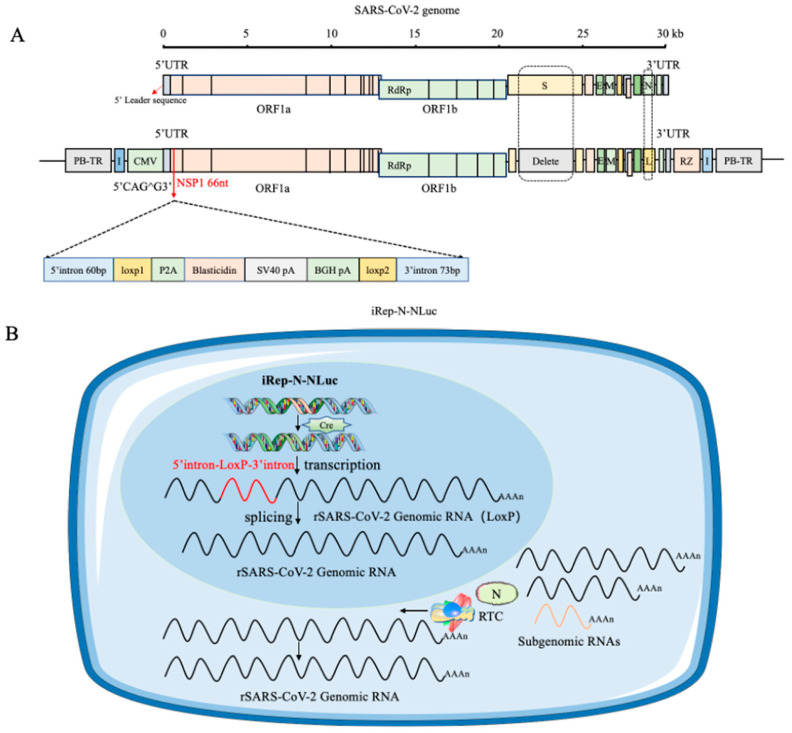
A system capable of replicating the SARS-CoV-2 virus was created by co-transfecting it with the N-protein. When a specific recombination event occurs using the *Cre*/*loxP*-system, viral replication is restored. (**A**) The recombinant SARS-CoV-2 replicon is structured as iRep-N-NLuc. A transcription stop cassette was integrated between nucleotide positions 66 and 67 of NSP1, specifically at the potential exon/exon boundary CAG66 to G67. The sequence inserted comprises the specified components in a particular order. PiggyBac 5′ TR and chicken β-globin insulators are positioned at the 5′ end of the CMV promoter and at the 3′ end of the HDV RZ region, respectively, surrounding the transcription unit. NLuc is represented by the letter L. (**B**) A schematic representation of the intracellular process of inducible iRep-N-NLuc replication within a cell model. Without Cre recombinase, the CMV promoter is solely responsible for the expression of the blasticidin resistance gene, which in turn blocks SARS-CoV-2 replication, thereby enabling the selection of stably integrated cell lines. Upon the induction of Cre recombinase in replicon cell lines, site-specific recombination deletes the transcription stop cassette at the DNA level. The CMV promoter subsequently initiates viral genome transcription. RNA splicing within the nucleus removes the last remaining LoxP site. The nucleus of the cell is represented in blue and the cytoplasm in light blue [6].

**Figure 4:**
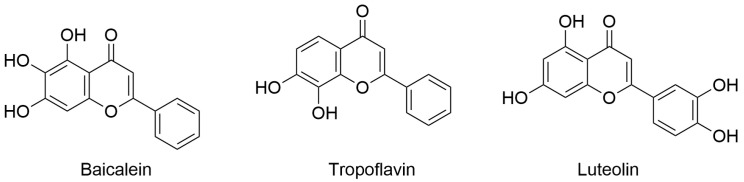
Flavones with anti-DENV activity. Chemical structure of baicalein (PubChem ID 5281605), tropoflavin (PubChem ID 1880), and luteolin (PubChem ID 5280445).

**Figure 5:**
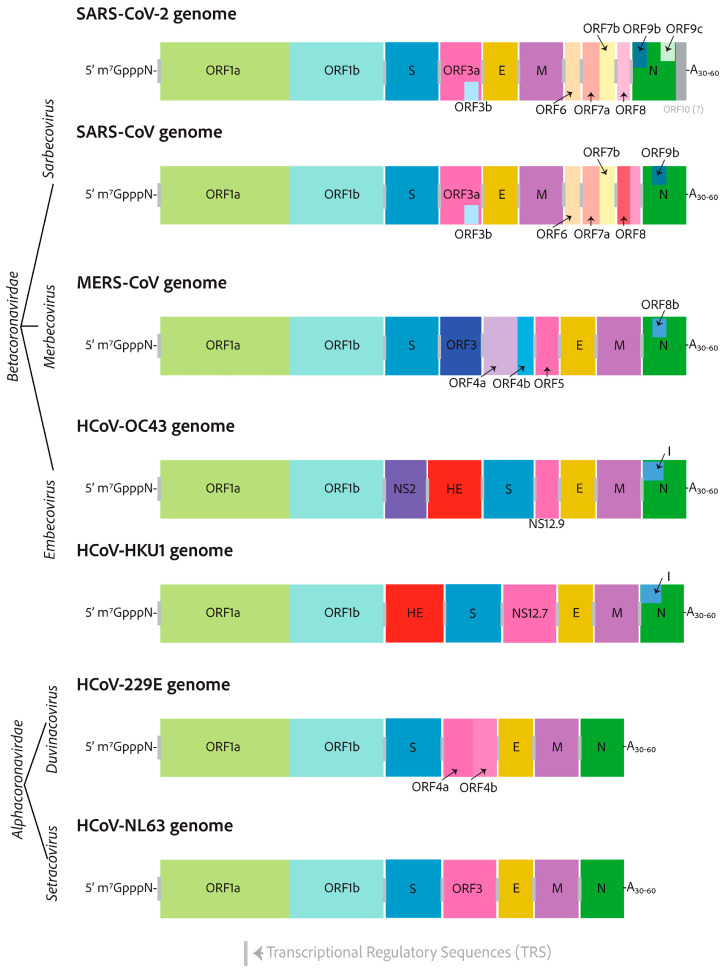
RNA genomes of seven clinically relevant coronaviruses. HCoV-229E and HCoV-NL63 (alphacoronaviruses), SARS-CoV-2, SARS-CoV, MERS-CoV, HCoV-OC43, and HCoV-HKU1 (betacoronaviruses). All coronavirus RNA genomes are capped with a 7-methylated-guanosine cap, which is linked by a 5′–5′ triphosphate bridge, a structure similar to that found in host cell mRNAs. Furthermore, the genomes contain a 3′ poly-A tail, which serves as a protective mechanism against host cell immune defenses, preventing mRNA degradation. The lengths of alphacoronaviruses are roughly 27 kilobases, whereas betacoronaviruses are approximately 30 kilobases in size and possess at their 3′-ends additional open reading frames [8].
